# Are depression, anxiety and poor mental health risk factors for knee pain? A systematic review

**DOI:** 10.1186/1471-2474-15-10

**Published:** 2014-01-09

**Authors:** Pyae P Phyomaung, Julia Dubowitz, Flavia M Cicuttini, Sanduni Fernando, Anita E Wluka, Paul Raaijmaakers, Yuanyuan Wang, Donna M Urquhart

**Affiliations:** 1 School of Public Health and Preventive Medicine, Department of Epidemiology and Preventive Medicine, Monash University, Alfred Hospital, Commercial Rd, Melbourne 3004, Victoria, Australia

**Keywords:** Psychosocial factors, General mental health, Depression, Anxiety, Knee pain, Osteoarthritis

## Abstract

**Background:**

While it is recognized that psychosocial factors are important in the development and progression of musculoskeletal pain and disability, no systematic review has specifically focused on examining the relationship between psychosocial factors and knee pain. We aimed to systematically review the evidence to determine whether psychosocial factors, specifically depression, anxiety and poor mental health, are risk factors for knee pain.

**Methods:**

Electronic searches of MEDLINE, EMBASE and PsycINFO were performed to identify relevant studies published up to August 2012 using MESH terms and keywords. We included studies that met a set of predefined criteria and two independent reviewers assessed the methodological quality of the selected studies. Due to the heterogeneity of the studies, a best evidence synthesis was performed.

**Results:**

Sixteen studies were included in the review, of which 9 were considered high quality. The study populations were heterogeneous in terms of diagnosis of knee pain. We found a strong level of evidence for a relationship between depression and knee pain, limited evidence for no relationship between anxiety and knee pain, and minimal evidence for no relationship between poor mental health and knee pain.

**Conclusions:**

Despite the heterogeneity of the included studies, these data show that depression plays a significant role in knee pain, and that a biopsychosocial approach to the management of this condition is integral to optimising outcomes for knee pain.

## Background

Knee pain is a widespread clinical problem, with almost half of those aged 50 and over reporting pain at the knee and 25% of these experiencing symptoms of a chronic nature [1]. The main underlying cause of knee pain is osteoarthritis (OA), a chronic joint disorder imposing significant health care burden [2]. With the advent of new methods for assessing joint structure, in particular non-invasive techniques such as magnetic resonance imaging (MRI), there has been increasing interest in factors associated with pain in knee OA. We recently showed that improvements in knee pain were associated with increased vastus medialis cross sectional area and beneficial structural changes at the knee including a reduction in loss of knee cartilage and in the rate of knee replacements [3]. While a number of factors are involved in structural change at the knee, these findings suggests that managing pain may be one factor that is important in reducing OA progression and that reducing pain may have long term structural benefits at the knee.

It is becoming increasingly evident that structural changes alone do not account for all musculoskeletal pain. Psychosocial factors have been shown to be predictors of pain and disability in a number of musculoskeletal conditions including chronic low back pain [4] and neck pain [5]. While two systematic reviews of prognostic factors for knee pain have specifically examined one or two psychosocial factors within a number of demographic, physical and patient-related factors [6-8], no systematic review has specifically focused on examining the relationship between psychosocial factors and knee pain. Moreover, the evidence from studies of knee pain is conflicting. While several cross-sectional studies have reported no association between depression and knee pain [8,9], others have reported depressive symptoms to be related to pain at the knee (Salaffi et al [10]; Wright [11]), Understanding the relationship between psychosocial factors and pain at the knee is important if we are to optimally manage knee conditions. The aim of this review was to systematically review the literature to determine whether depression, anxiety and poor mental health are risk factors for knee pain.

## Methods

A systematic review was conducted according to 2009 PRISMA statement [12].

### Data sources and search strategy

An initial search of MEDLINE, EMBASE and PsycINFO was performed to identify studies that examined the relationship between psychosocial factors and knee pain using the MeSH terms; ‘knee pain’, knee osteoarthritis,’ and the keywords: ‘knee’, ‘osteoarthritis’, ‘pain’, ‘psychosocial’, ‘psychosomatic’, ‘psychological,’ ‘psychophysiologic’. The search was limited to human studies of adults published in the English language.

The results of this search showed that there were a large number of studies in this field investigating a broad range of psychosocial factors, with a considerable number focussing on the role of depression, anxiety and general mental health. Thus, a second search was undertaken to identify studies on these three psychosocial factors. All extracted studies were independently reviewed by two reviewers (SF, PP) to identify relevant articles. Where the reviewers disagreed and could not achieve consensus, a third reviewer (DU) gave a final judgement. The reference lists of all included studies were also examined to find any additional key studies.

### Inclusion and exclusion criteria

Studies were included if they examined depression, anxiety and poor mental health as potential risk factors for knee pain, or trials which investigated the effect of interventions addressing these psychological factors on knee pain. Studies on knee pain were included whether or not knee OA was specified.

**Exclusion criteria**: (1) Studies that did not separate knee pain from pain in other regions such as the hip and back; (2) Studies investigating the reverse outcome (i.e. the effect of pain on psychosocial health); (3) Studies that did not focus on pain at the knee; (4) Study participants who had rheumatologic conditions or other associated medical conditions affecting joints; and (5) Study populations who had undergone knee surgery.

### Data extraction

Data on the characteristics of the included studies were extracted, including: (1) Study design (including cross-sectional, case-control and cohort studies, and randomised control trials); study population; number of participants; mean age and percentage of female participants; definition of OA previous knee injury; (2) Method of assessment of psychosocial factors (depression, anxiety and poor mental health); (3) Outcome measures; assessment of knee pain and (4) Study results.

### Methodological quality assessment

The methodological quality of each study was assessed independently by two reviewers (JD, SF) using standard criteria adapted from Lievense et al [13] (Table 1). These criteria allow the quality of cross-sectional, case-control and cohort studies to be assessed. Only relevant criteria for each study type were included in calculations of the total and percentage mean quality score. Scores were compared between raters and a consensus score was obtained by agreement for each study. Any study which obtained a score above the mean was considered to be of high quality.

**Table 1:** Criteria used to assess the methodological quality of selected cohort and cross-sectional studies

**Item**	**Criterion**	**Study type**
*Study population*		
1	Selection before disease was present or at uniform point	CH/CC/CS
2	Cases and controls were drawn from the same population	CC
3	Participation rate ≥80% for cases/cohort	CH/CC/CS
4	Participation rate ≥80% for controls	CC
5	Sufficient description of baseline characteristics	CH/CC/CS
*Assessment of risk factor*		
6	Psychosocial assessment was blinded	CH/CC/CS
7	Psychosocial factors were measured identical for cases and controls	CC
8	Psychosocial factors were assessed prior to the outcome	CH/CC/CS
*Assessment of outcome*		
9	Knee OA/pain was assessed identical in studied population	CH/CC/CS
10	Presence of knee OA/pain was assessed reproducibly	CH/CC/CS
11	Presence of knee OA/pain was assessed according to standard definitions	CH/CC/CS
*Study design*		
12	Prospective design was used	CH/CC/CS
13	Follow up time ≥2 years	CH
14	Withdrawals ≤20%	CH
*Analysis and data presentation*		
15	Appropriate analysis techniques were used	CH/CC/CS
16	Adjusted for at least age and sex	CH/CC/CS

CH, Applicable to cohort studies; *CC*, Applicable to case-control studies; *CS*, Applicable to cross-sectional studies; *OA*, Osteoarthritis.

As the Lievense et al [13] did not include criteria specific to the methodological assessment of randomised controlled trials (RCTs), the PEDro scale was used for the quality assessment of RCTs [14]. The PEDro scale rates 11 aspects of methodological quality of RCTs as being either absent or present (Table 2). As the first item (eligibility criteria) is not scored, the total score ranges from 0 to 10. Studies that obtain a score of <6 points are considered to have low quality, while those with a score ≥6 points are reported to be of high quality.

**Table 2:** The PEDro Scale Criteria used to assess the methodological quality of selected randomised control trials

	**Yes**	**No**	**Where/ comments**
1. Eligibility criteria were specified			
2. Subjects were randomly allocated to groups (in a crossover study, subjects were randomly allocated an order in which treatments were received)			
3. Allocation was concealed			
4. The groups were similar at baseline regarding the most important prognostic indicators			
5. There was blinding of all subjects			
6. There was blinding of all therapists who administered the therapy			
7. There was blinding of all assessors who measured at least one key outcome			
8. Measures of at least one key outcome were obtained from more than 85% of the subjects initially allocated to groups			
9. All subjects for whom outcome measures were available received the treatment or control condition as allocated or, where this was not the case, data for at least one key outcome was analysed by “intention to treat”			
10. The results of between-group statistical comparisons are reported for at least one key outcome			
11. The study provides both point measures and measures of variability for at least one key outcome			
TOTAL (checked excluding eligibility criteria specified):			

### Data synthesis

Due to heterogeneity in the methodology between studies, the decision was made to use a best evidence synthesis to summarise the data (Table 3). Studies were ranked according to their design, with cohort studies considered to be a higher level of evidence than case control and cross-sectional studies. The level of evidence of studies was determined in conjunction with the quality score calculated for each study. Where we identified only a few high quality cross-sectional studies with consistent findings and these did not fit one of the best evidence synthesis levels of evidence (Table 3), we described the evidence as ‘minimal’.

**Table 3:** **Criteria list for determining the level of evidence for best evidence synthesis, adapted from Lievense et al (2001)**[13]

**Level of evidence**	**Criteria for inclusion in best evidence synthesis**
Strong evidence	generally consistent findings in:
	o multiple high quality cohort studies
Moderate evidence	generally consistent findings in:
	o 1 high quality cohort study & > 2 high quality case-control studies
	o > 3 high quality case-control studies
Limited evidence	generally consistent findings in:
	o single cohort study
	o 1 or 2 case-control studies or
	o multiple cross-sectional studies
Conflicting evidence	inconsistent findings in <75% of the trials
No evidence	No studies could be found

## Results

### Identification and selection of the literature

**Figure 1:**
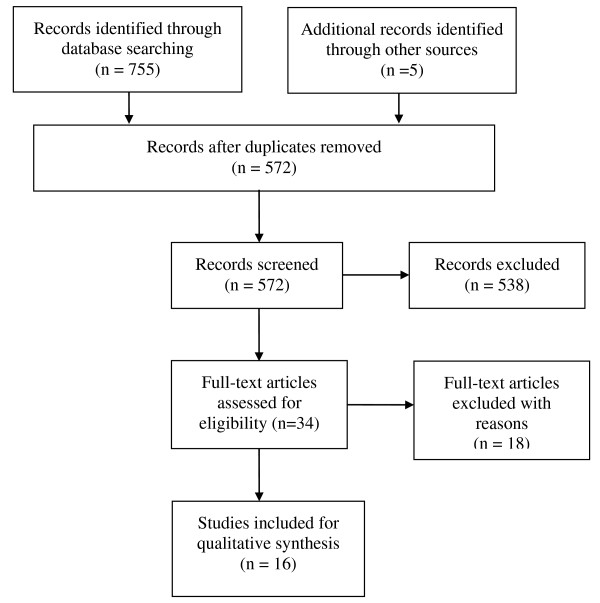
Flow diagram of included and excluded studies according to the PRISMA statement.

Of the 755 studies that were identified from our electronic database search, 34 were potentially eligible for inclusion (Figure 1). The full text of these studies was obtained and a further 18 were excluded as they examined self-management practices [15], the pain experience [16], ethnicity [17], musculoskeletal pain (not specifically knee pain) [18-21], walking speed [22], whole body pain intensity [23,24], OA in general (not specifically knee OA) [25-27], prediction of somatisation disorder [28] and the effect of pain on psychological health [29]. Of the three remaining studies, one was a validation study [30], the second was a literature review [31] and the third was a RCT which assessed patients with hip and knee OA together [32].

### Characteristics of included studies

Sixteen studies were included (Table 4). Of these, 10 were cross-sectional [8-11,33-38], 1 was nested case-control study [39], 2 were cohort studies [12,40] and 3 were randomised controlled trials [41-43]. Nine studies were undertaken in the USA [8,11,34,35,38,40-42,44], 1 in the Netherlands [9], 2 in England [33,39], and 1 each in Italy [10], Egypt [43], New Zealand [36], and Japan [37].

**Table 4:** Characteristics of included studies

**Author (country, year)**	**Study population**	**No. of participants**	**Age (years)**	**Definition of OA**	**Previous knee injury**	**Pain assessment**	**Psychosocial factor assessment**	**Quality score**
		**(% women)**	**mean ± SD (range)**					
***Cross-sectional Studies***								
O’Reilly (England, 1998)	Community participants registered at two general practices and aged 40–70 years	3323 (NA)	NA (range: 40–75)	NA	NA	Questions regarding knee pain on most days for at least a month (in the past year)	General mental health: Short Form 36 (SF36) subscale	45
Creamer (USA, 1999)	Recruited from the Baltimore Longitudinal Study of Aging; community-based individuals >40 years	374 (32)	Men:	NA	NA	Knee pain: National Health and Nutrition Examination Survey	Anxiety: Arthritis Impact Measurement Scales (AIMS)	55
			63.8 ±0.80					
			Women: 62.8 ±1.08					
							Depression: AIMS	
Harcombe (New Zealand, 2010)	Randomly selected nurses, postal workers and office workers using computers	443 (NA)	NA (range: 20–59)	NA	NA	Self-reported knee pain lasting for more than a day in the month before the survey	General Mental health: Mental Health Inventory-5 (MHI-5)	73
Matsudaira (Japan, 2011)	Nurses, office workers, sales/marketing personnel and transportation operatives	2290 (32)	NA (range: 19–64)	NA	NA	Self-reported knee pain in the past month and past year	General Mental health: SF36 subscale	82
Creamer (USA, 1999)	Outpatients with prior physician diagnosis of knee OA and current knee pain	68 (69.1)	65.8 ± 10.4	American College of Rheumatology clinical criteria	Excluded if previous total knee replacement	Knee Pain and Severity: WOMAC, VAS, MPQ	Depression: Centre for Epidemiological Studies Depression Scale (CES-D)	55
							Anxiety: State-Trait Anxiety Inventory (STAI)	
Davis (USA, 1992)	Study sample from NHANES I survey, aged 45–74 years, who had knee OA and knee pain	4056 (52)	(45–74)	OA based on radiographic criteria using the Atlas of Standard Radiographs of Arthritis.	NA	Knee pain on most days lasting one month in the past year or pain on active or passive motion during the examination	General Mental Health: NHANES General Wellbeing Index	45
Salaffi (Italy, 1991)	61 participants from outpatient clinic of a Rheumatic Disease Unit with symptomatic knee OA	61 (100)	63.5 ± 7.3	American College of Rheumatology clinical criteria	NA	Knee Pain: MPQ and Visual Analogue Scale	Depression: Zung Depression Inventory	45
							Anxiety: Zung Anxiety Inventory	
van Baar	Participants presenting to their GPs with hip and knee OA	Hip OA: 73 (71.2)	Hip OA:	American College of Rheumatology clinical criteria	Excluded if pathology explained the complaints	Severity of knee pain: Visual Analogue Scale	Anxiety and Depression: IRGL questionnaire	64
(The Netherlands, 1998)		Knee OA: 112 (88.4)	67.7 ± 8.7					
			Knee OA: 69.3 ± 8.1					
Pells (USA, 2008)	Subjects with knee OA recruited through Rheumatology, Orthopaedic Surgery, and Pain Management clinics	174 (82)	57.7 ± 9.8	American College of Rheumatology clinical criteria	NA	Knee pain: AIMS	Depression and Anxiety: Psychological Disability subscale of AIMS	64
Wright (USA, 2008)	Participants from the KNEE study, aged 35–64 years; pain on ≥4 days a week	275	NA (range 35–64)	American College of Rheumatology clinical criteria	Excluded if have inflammatory arthritis, previous knee surgery, Kellgren and Lawrence grade III-IV	Pain: WOMAC pain subscale	Depressive symptoms: CES-D	82
						Pain composite: pain assessments taken after physical function tests in pre-baseline assessment		
							General mental health (Vitality): subscale of the SF-36	
***Nested case–control studies***								
Peat (United Kingdom, 2009)	Both cases and control are recruited from the Clinical Assessment Study of the Knee	285 (55)	Cases:	NA	Previous knee surgery n (%): 26 (9.1)	Characteristic pain intensity: Chronic Pain Grade	Anxiety and depression: Hospital Anxiety and Depression Scale	79
			66.3 ± 9.2					
			Controls: 64.6 ± 8.2					
						Pain extent: areas of pain experienced in previous month shaded on whole-body manikin		
						Night pain: single item on WOMAC		
***Longitudinal Studies***								
Piva (USA, 2009)	Subjects diagnosed with patella-femoral pain syndrome (PFPS) recruited from rehabilitation clinics	74 (52)	29 ± 9	NA	Excluded if previous patellar dislocation, knee surgery past 2 years, ligamentous injury or laxity, internal derangement	Knee pain intensity measured using 11-point numerical pain rating scale (NPRS)	Anxiety: Beck Anxiety Index	85
Riddle (USA, 2011)	Community based recruitment through 4 teaching hospitals from different states (Osteoarthritis initiative study)	3405 (59.1%)	60.62 ±9.04	Modified Kellgren and Lawrence Knee OA	NA	Knee Pain: WOMAC pain scale	General mental health: SF-12 Mental Component Summary (MCS)	92
						Disability: WOMAC disability scale		
							Depression: 20-item CES-D	
***Randomised controlled trials***								
Chappell	Male and female outpatients ≥ 40 years of age. Recruitment by clinical sites	Antidepressant (intervention)= 128(69.5%)	Antidepressant= 63.2 ± 8.8	American College of Rheumatology clinical criteria	Excluded patients with invasive therapies to the index knee during the past 3 months or previous joint replacement anytime	Knee Pain: Brief Pain Inventory (BPI); WOMAC pain and stiffness subscales	Depression: Beck Depression Inventory-II (BDI-II);	8*
(USA, 2011)								
			Placebo=					
		Placebo Control= 128(83.6%)	61.9 ± 9.2					
	in Canada, Greece, Russia, Sweden, and the USA by					Perceived improvement: Clinical Global Impressions of Severity (CGI-S)	Hospital Anxiety and Depression Scale anxiety subscale	
	general practitioner and rheumatologists							
							(HADS-A)	
Chappell	Outpatients of ≥40 years male and female with pain for 14 days of each month for 3 months before study entry, with a mean score on the 24-h average pain score (0–10) using the average of daily ratings from visit 1 to visit 2	Antidepressant	Antidepressant= 62.1 ± 9.6	American College of Rheumatology clinical criteria	Excluded patients with previous invasive knee surgery, arthroscopy and joint replacement	Knee Pain: Weekly 24-h worst pain; WOMAC pain subscale	Depression: Beck Depression Inventory-II	9*
(USA, 2009)		(intervention)= 111 (63.1%)						
			Placebo=					
		Placebo Control	62.5 ± 9.3					
		120 (67.5%)					Hospital	
						Severity: BPI-S, Brief Pain Inventory-Severity; CGI-S, Clinical Global Impressions of Severity	Anxiety and Depression Scale (HADS)	
Abou-Raya	Aged 65 years and above attending the outpatient clinic	Antidepressant	Antidepressant= 68.9 ± 6.2	American College of Rheumatology clinical criteria Radiographic criteria K/L grade I–III	NA	Knee Pain: Visual analogue pain scale	Depression: Geriatric depression scale	10*
(Egypt, 2012)		(intervention)= 144 (84%)						
			Placebo= 68.5 ± 5.8					
		Placebo Control 144 (84%)				WOMAC pain score		

***NHANES***, National Health and Nutritional Examination Survey; ***PFS***, Physical Functioning Score; ***WOMAC***, Western Ontario and McMaster University Osteoarthritis Index; ***PCI***, Pain Coping Inventory; ***4DSQ***, Four Dimensional Symptom Questionnaire; ***CES-D***, Centre for Epidemiological Studies Depression Scale; ***QOL***, Quality of Life; ***SF-36***, Short-Form-36 Health Survey; ***SSS***, Social Support Scale; ***VAS***, Visual Analogue Scale; ***OA***, osteoarthritis; ***K/L scale***, Kellgren and Lawrence Atlas of Standard Radiographs of Arthritis; ***WOMAC***, Western Ontario and McMaster University Arthritis Index; ***MPQ***, McGill Pain Questionnaire; ***AIMS***, Arthritis Impact Measurement Scales; ***ACR***, American College of Rheumatology; ***NA***, not available; ***PFS***, Physical Functioning Scale; ***IRGL***, Invloed van Reuma op Gezondheid en Leefwijze (Dutch version of the Arthritis Impact Measurement Scale). *Indicates quality scores for RCTs as per the PEDro scale.

Participants were recruited or participant data were obtained from: outpatient and rehabilitation clinics in 7 studies [8,10,34,40-43], GP clinics in 2 studies [9,33], previous studies, including the Baltimore Longitudinal Study of Aging (community-based), NHANES survey, KNEE study, and the Clinical Assessment Study of the Knee, in 4 studies [11,35,38,39], various occupational groups including nurses, postal and office workers, sales/marketing personnel and transportation operatives in 2 studies [36,37] and community and teaching hospitals in 1 study [44]. The mean age of the subjects ranged from 29.0 to 69.3 years with the percentage of females varying from 32 to 100 percent. One study excluded participants due to previous injury [40] and 6 studies as a result of previous surgery [11,34,39-42].

#### Diagnosis of OA in study participants

Various methods were used to identify OA in participants. Of the 10 studies that specified how the diagnosis of OA was confirmed; 8 studies used criteria specified by the American College of Rheumatology [8-11,34,41-43], 1 used x-rays graded according to the modified Kellgren/Lawrence score [44], and 1 used their own four point radiographic assessment score [38].

#### Assessment of pain

A number of scales were used to assess pain. The most common scales used were; the Western Ontario and McMaster Universities Arthritis Index (WOMAC) in 7 studies [11,34,39,41-44], the Visual Analogue Scale in 4 studies [9,10,35,43] and question(s) regarding the prevalence of pain over the past month and/or year in 4 studies [33,36-38]. Other pain scales used were the Chronic Pain Grade Scale, McGill Pain Questionnaire and the National Health and Nutritional Examination Survey.

#### Assessment of psychosocial factors

The assessment of depression, anxiety and general mental health was performed using a variety of methods. Depression was assessed by 7 different methods, including the Centre for Epidemiological Studies Depression scales [11,34,44], Hospital Anxiety and Depression Scale [39,41,42] and Arthritis Impact Measurement Scales [8,35]. Anxiety was assessed using 5 different scales across 6 studies; Arthritis Impact Measurement Scales (both English and Dutch version) [9,35], Hospital Anxiety and Depression Scale [39], Beck Anxiety Index [40], Zung Anxiety Inventory [10], and the State-Trait Anxiety Inventory [34]. General mental health was assessed using 3 different questionnaires; the Short Form-36 [33][37] the Mental Health Inventory [36] and the NHANES General Wellbeing Index [38].

#### Methodological quality assessment

The mean methodological quality score of the included observational studies was 67%, with scores ranging from 45% to 92% Additional file 1. Six of the 13 observational studies were considered to be of high quality (according to the Lievense criteria), as they were given a quality score above the mean. All three of the RCTs were considered high quality as they scored greater than 6 on the PEDro scale.

Analysis of the quality scores and criteria revealed that most studies achieved high scores on selection of participants with disease at uniform point (criteria 1), identical assessment of outcome (criteria 9), sufficient description of baseline characteristics (criteria 5), analysis technique (criteria 15), and adjustment for age and sex (criteria 16). However, a number of studies scored poorly on blinded assessment of the psychosocial risk factor (criteria 6), assessment of the risk factor prior to outcome (criteria 8) and reproducible assessment of outcome (criteria 10). Only 5 studies used prospective designs and of these, 2 were cohort studies and 3 were RCTs.

### Relationship between depression and knee pain

Six cross-sectional studies [8-11,34,35], one nested case-control study [39], one longitudinal study [44], and three RCTs assessed the relationship between depression and knee pain [41-43] (Table 5).

**Table 5:** Studies examining the relationship between depression and knee pain

**Author (year)**	**Study design**	**Assessment of depression**	**Assessment of pain pain/OA**	**Results**	**Conclusion**	**Quality score**
Creamer (1999- Baltimore study)	Cross-sectional	Arthritis Impact Measurement Scales (AIMS) Questionnaire (Depression subscale)	Pain on most days for at least one month (National Health and Nutrition Examination Survey (NHANES-1))	Pain reporting was not related to depression (statistics not provided).	Depression was not associated with knee pain.	55
				Depression scores were higher in subjects reporting ‘ever’ pain in the presence of normal radiographs than in those without reported knee pain (1.70 ± 0.27 versus 1.16 ± 0.09), but this was not statistically significant (*P=* 0.06).		
Creamer (1999)	Cross-sectional	Centre for Epidemiological Studies Depression Scale (CES-D)	Pain Severity	Unadjusted Correlations: MPQ: r= 0.31 (p < 0.05).	There was no association between depression and pain severity after adjustment.	55
			(WOMAC, Visual Analogue Scale,	VAS: r= 0.19 (NS)		
			McGill Pain Questionnaire (MPQ))	WOMAC: r= 0.15 (NS)		
				In the stepwise regression models after adjustment, depression did not remain in the model.		
Salaffi (1991)	Cross-sectional	Zung Depression Inventory	Pain	Stepwise multiple regression:	Depression was found to be associated with the pain experience.	45
			(McGill Pain Questionnaire (MPQ), Visual Analogue Scale (VAS))			
				MPQ: R= 0.41; t= 2.99; p < 0.01		
				VAS R= 0.39; t= 2.77; p < 0.01		
van Baar (1998)	Cross-sectional	IRGL Questionnaire	Severity of pain: Visual Analogue Scale	Bivariate Correlation:	Depression was not associated with knee pain.	64
				Knee pain: r= 0.28 p ≤ 0.01		
				Regression Analysis: NS (not remain in the model)		
Wright (2008)	Cross-sectional	CES-D	WOMAC pain scale	WOMAC: mean= 17.76 ± 14.47	There was an association between knee pain and depressive symptoms.	82
		Psychological Disability subscale of AIMS				
				Depressive Sx: mean= 1.80 ± 2.79		
				Neuroticism: mean= 2.26 ± 0.59		
				Negative affect: mean= 1.67 ± 0.51		
				Correlation between pain and depressive Sx: r= 0.21; *p < 0.01*		
				Correlation between pain and negative affect: r= 0.15; *p < 0.05*		
Pells (2008)	Cross-sectional	Psychological Disability subscale of AIMS	AIMS	Correlation between psychosocial disability and AIMS pain scale: r= 0.24; p < 0.01.	Pain did not demonstrate an association with psychological disability.	64
				Multiple regression: NS		
Peat (2009)	Nested case-controlled	Hospital Anxiety and Depression Scale	Characteristic pain intensity: Chronic Pain Grade	Mean difference (95% CI) of depression between cases and controls at 18 months: 2.2 (1.2 to 3.1)	Substantial deterioration of knee pain is accompanied by an increase in depressive symptoms.	79
			Pain extent: areas of pain experienced in previous month shaded on whole-body manikin			
				Cases were subjects who had mild knee pain at study entry and become severe at 18 months follow up.		
			Night pain: single item on WOMAC	Controls were subjects who still had mild knee pain at 18 months follow up and were selected from similar cohort as cases).		
Riddle (2011)	Longitudinal Cohort Study	20-item CES-D	Knee Pain: WOMAC pain scale	dichotomised CES-D score (≥16)	Baseline depression is the most consistent psychological predictor of yearly worsening of pain. Association exists after adjusting for confounding variables.	92
			Disability: WOMAC disability scale	Univariate analysis: WOMAC Pain: Estimate (95% CI)= 0.36 (0.16 to 0.56); p < 0.001		
				Multivariate analysis: WOMAC Pain: Estimate (95% CI)= 0.59 (0.18 to 1.01); p= 0.005		
Chappell	Randomised Controlled Trial(RCT) investigating the effect of antidepressant (Duloxetine) on knee OA	Beck Depression Inventory-II (BDI-II) Hospital Anxiety and Depression Scale anxiety subscale (HADS-A)	Knee Pain: Brief Pain Inventory (BPI); WOMAC pain and stiffness subscales Perceived improvement: Clinical Global Impressions of Severity (CGI-S)	Mean change in pain score from baseline (at 13 weeks)	Treatment with duloxetine 60 to 120 mg was associated with significant pain reduction in patients with pain due to knee OA.	8*
(USA, 2011)						
				BPI average pain (% response)		
				≥30%= 65.3 (antidepressant group= I) & 44.1 (placebo= C); p ≤ 0.001		
				WOMAC: -13.74 (I) -17.51 (C); p ≤0.05		
				CGI-S: -0.40 (I) & -0.70(C); p ≤ 0.01		
Chappell	RCT investigating the effect of antidepressant (Duloxetine) on knee OA	Beck Depression Inventory-II	Knee Pain: Weekly 24-h worst pain; WOMAC pain subscale	Mean change (SD) in pain score from baseline (at 13 weeks)	Duloxetine demonstrated statistically significant pain reduction compared with placebo.	9*
(USA, 2009)						
		Hospital Anxiety and Depression Scale (HADS)				
				BPI-S(Average pain): –2.82 ±0.21(C) –1.85 ± 0.21(C); p < .001		
			Severity: BPI-S, Brief Pain Inventory-Severity; CGI-S, Clinical Global Impressions of Severity			
				WOMAC: –4.64 ± 0.35 (I)		
				−3.24 ± 0.35(C); p= 0.003		
				CGI-S: -0.65 ±0.08(I) & –0.29 ± 0.08(C); p= 0.001		
Abou-Raya	RCT investigating the effect of antidepressant (Duloxetine) on knee OA	Geriatric depression scale	Knee Pain Visual analogue pain scale; WOMAC pain score	WOMAC pain score	Duloxetine has a dual beneficial effect of improving depression and pain symptoms in older adults with knee OA.	10*
(Egypt, 2012)				(0–20): Mean (SD)		
				At baseline: Intervention - 9.1(4.6)		
				Placebo - 8.9(5.1); p= 0.44		
				At 16 weeks : Intervention - 6.0 (4.1) Placebo - 8.4 (5.4); p= 0.05		

***NHANES***, National Health and Nutritional Examination Survey; ***PFS***, Physical Functioning Score; ***WOMAC***, Western Ontario and McMaster University Osteoarthritis Index; ***PCI***, Pain Coping Inventory; ***4DSQ***, Four Dimensional Symptom Questionnaire; ***CES-D***, Centre for Epidemiological Studies Depression Scale; ***QOL***, Quality of Life; ***SF-36***, Short-Form-36 Health Survey; **SSS** - Social Support Scale; ***VAS***, Visual Analogue Scale; ***OA***, osteoarthritis; ***K/L scale***, Kellgren and Lawrence Atlas of Standard Radiographs of Arthritis; ***WOMAC***, Western Ontario and McMaster University Arthritis Index; ***MPQ***, McGill Pain Questionnaire; ***AIMS***, Arthritis Impact Measurement Scales; ***ACR***, American College of Rheumatology; ***NA***, not available; ***PFS***, Physical Functioning Scale; ***IRGL***, Invloed van Reuma op Gezondheid en Leefwijze (Dutch version of the Arthritis Impact Measurement Scale) *Indicates quality scores for RCTs as per the PEDro scale.

Of the 6 cross-sectional studies, only one was considered high quality. The high quality study found a significant association between knee pain and depressive symptoms (r= 0.21, p < 0.01) [11]. Of the 5 low quality studies [8,10,34,35], only 1 study found a significant association between depression and knee pain (r= 0.41, p < 0.01) [38].

The nested case-control study, which was of high quality, found that substantial deterioration of knee pain was accompanied by higher frequency of depressive symptoms among cases (those participants experiencing progression of pain intensity from mild to severe) compared to controls (those not experiencing progression of pain) [39]. The single longitudinal cohort study was also of high quality and found the presence of baseline depressive symptoms was the most consistent psychological predictor of worsening pain over the follow up period (Coefficient (95% CI): 0.59 (0.18, 1.01), p= 0.05) [44].

The three RCTs, all rated as high quality, examined the effect of SNRI (Serotonin Noradrenalin Reuptake Inhibitor) antidepressant on change in pain intensity among knee OA patients [41-43]. All showed that treatment with antidepressant medication was associated with significant pain reduction and that SNRI antidepressants (duloxetine) reduced pain compared to placebo. One RCT [43] showed that older adults with knee OA treated for 16 weeks with duloxetine (SNRI) had significantly greater pain reduction than those treated with placebo. Subgroup analyses of two of the trials showed that the duration of pain and severity of OA did not affect the efficacy of treatment [41,42].

### Relationship between anxiety and knee pain

Of the 6 studies that examined the relationship between anxiety and knee pain, 4 were cross-sectional studies [9,10,34,35], one was a nested case-control study [39] and one was a longitudinal cohort study [40] (Table 6). The cross-sectional studies were of low quality, while the nested case-control study [39] and the longitudinal cohort study [40] were of high quality. The low quality cross-sectional studies reported mixed results [9,10,34,35], while the high quality studies reported no significant association between anxiety and knee pain [39,40].

**Table 6:** Studies examining the relationship between anxiety and knee pain

**Author (year)**	**Study design**	**Assessment of anxiety**	**Assessment of pain**	**Results**	**Conclusion**	**Quality score**
Creamer (1999 – Baltimore study)	Cross-sectional	Arthritis Impact Measurement Scales (AIMS) Questionnaire: (Anxiety subscale)	Pain on most days for at least one month (NHANES-1)	Women reporting having knee pain had higher anxiety than those reporting never having knee pain (3.06 ± 0.26 vs 2.35 ± 0.17, p= 0.025).	Anxiety was associated with pain in women, but not men.	55
				Pain reporting was not related to anxiety in men (data not shown).	Women reporting knee pain, in the absence of radiographic osteoarthritis, had higher anxiety scores than those without pain.	
				Analysis stratified by radiographic severity. It showed that differences in anxiety were confined to subjects reporting knee pain in the absence of radiographic change (i.e., KL grade 0) (statistics not available).		
Creamer (1999)	Cross-sectional	State-Trait Anxiety Inventory (STAI)	Pain Severity	MPQ: r= 0.30 (p < 0.05).	Anxiety was not found to be associated with pain in patients with knee OA.	55
			(WOMAC, Visual Analogue Scale,	VAS: r= 0.19 (NS)		
				WOMAC: r= 0.23 (NS)		
			McGill Pain Questionnaire (MPQ))	In the stepwise regression models after adjustment, anxiety did not remain.		
Salaffi (1991)	Cross-sectional	Zung Anxiety Inventory	Pain	Stepwise multiple regression:	Anxiety was found to be related to pain.	45
			(McGill Pain Questionnaire (MPQ), Visual Analogue Scale (VAS))	MPQ: R= 0.19; t= 2.245 p < 0.05		
				VAS: R= 0.21; t= 2.88; p < 0.01		
Van Baar (1998)	Cross-sectional	IRGL Questionnaire	Severity of pain: Visual Analogue Scale	Bivariate Correlation:	Anxiety was not associated with knee pain although there was bivariate correlation between anxiety and pain.	64
				Knee pain: r= 0.30 p ≤ 0.01		
				Regression Analysis: NS		
Peat (2009)	Nested case control	Hospital Anxiety and Depression Scale	Characteristic pain intensity: Chronic Pain Grade	Mean difference (95% CI) of anxiety between cases and controls at 18 months: 1.0	There was no significant association between knee pain and perceived anxiety.	79
			Pain extent: areas of pain experienced in previous month shaded on whole-body manikin	(−0.2 to 2.3)		
			Night pain: single item on WOMAC			
Piva (2009)	Longitudinal	Beck Anxiety Index	11 point Numerical Pain Rating Scale (NPRS)	Correlation with anxiety	There was no significant association between anxiety and pain.	85
				NPRS: r= 0.34; P ≤ 0.01		
				Forward Multiple Regression- Not significant		

***NHANES***, National Health and Nutritional Examination Survey; ***PFS***, Physical Functioning Score; ***WOMAC***, Western Ontario and McMaster University Osteoarthritis Index; ***PCI***, Pain Coping Inventory; ***4DSQ***, Four Dimensional Symptom Questionnaire; ***CES-D***, Centre for Epidemiological Studies Depression Scale; ***QOL***, Quality of Life; ***SF-36***, Short-Form-36 Health Survey; ***SSS***, Social Support Scale; ***VAS***, Visual Analogue Scale; ***OA***, osteoarthritis; ***K/L scale***, Kellgren and Lawrence Atlas of Standard Radiographs of Arthritis; ***WOMAC***, Western Ontario and McMaster University Arthritis Index; ***MPQ***, McGill Pain Questionnaire; ***AIMS***, Arthritis Impact Measurement Scales; ***ACR***, American College of Rheumatology; ***NA***, not available; ***PFS***, Physical Functioning Scale; ***IRGL***, Invloed van Reuma op Gezondheid en Leefwijze (Dutch version of the Arthritis Impact Measurement Scale).

### Relationship between poor mental health and knee pain

Of the 4 cross-sectional studies examining the relationship between poor mental health and knee pain [33,36-38], 2 were of high quality [36,37] (Table 7). In contrast to the low quality studies that found a significant association between poor mental health and knee pain, both high quality studies found no significant association.

**Table 7:** Studies examining the relationship between poor mental health and knee pain

**Author (year)**	**Study design**	**Assessment of general mental health**	**Assessment of pain**	**Results**	**Conclusion**	**Quality score**
O’Reilly (1998)	Cross-sectional	SF-36 Questionnaire – Mental Health Component	Knee pain on most days for at least a month (in the past year)	Mental health score (<61): OR: 2.1 95% CI: 1.7-2.6	Lower mental health scores were associated with increased odds of knee pain.	45
				Knee pain: Median (IQR): 72(56–84)		
				No knee pain: Median (IQR): 76(64–88). P < 0.001		
Matsudaira (2011)	Cross-sectional	SF36 subscale	Self reported knee pain in past month or in the past year	Knee pain and mental health: Not significant (Data not provided)	There was no association found between knee pain and general mental health.	82
Harcombe (2010)	Cross-sectional	Mental Health Inventory-5 (MHI-5)	Self-reported knee pain lasting for more than a day in the month	Knee pain and mental health: OR (95% CI)= 0.96 (0.90 to 1.02); p value=0.194	There was no association between self-reported knee pain and mental health.	73
			Standardised Nordic Questionnaires for MSDs and Brief Symptom Inventory diagram showing the area of the body			
Davis (1992)	Cross-sectional	Psychological Wellbeing: NHANES General Wellbeing Index	Pain on most days lasting one month in the past year or knee pain on active or passive motion during the examination	Psychological wellbeing (score ≤70 & reference group >94)	Psychological wellbeing was associated with knee pain among participants with and without radiographic OA.	45
				OA and No OA: OR (95% CI)= 1.4 (1.0 to 2.0)		
				OA ± Pain: OR (95% CI)= 3.7 (1.8 to 7.6)		
				Pain ± OA: OR (95% CI)= 3.2 (2.1 to 5.0)		

***NHANES***, National Health and Nutritional Examination Survey; ***PFS***, Physical Functioning Score; ***WOMAC***, Western Ontario and McMaster University Osteoarthritis Index; ***PCI***, Pain Coping Inventory; ***4DSQ***, Four Dimensional Symptom Questionnaire; ***CES-D***, Centre for Epidemiological Studies Depression Scale; ***QOL***, Quality of Life; ***SF-36***, Short-Form-36 Health Survey; ***SSS***, Social Support Scale; ***VAS***, Visual Analogue Scale; ***OA***, osteoarthritis; ***K/L scale***, Kellgren and Lawrence Atlas of Standard Radiographs of Arthritis; ***WOMAC***, Western Ontario and McMaster University Arthritis Index; ***MPQ***, McGill Pain Questionnaire; ***AIMS***, Arthritis Impact Measurement Scales; ***ACR***, American College of Rheumatology; ***NA***, not available; ***PFS***, Physical Functioning Scale; ***IRGL***, Invloed van Reuma op Gezondheid en Leefwijze (Dutch version of the Arthritis Impact Measurement Scale).

### Best evidence synthesis

Due to the heterogeneity of the study designs, a best evidence synthesis was performed using studies classified as being of high quality. A study was considered to be of high quality if the methodological quality score was greater than 67%.

#### Depression and knee pain

One cross-sectional study, one nested case-control study, one longitudinal study and three RCTs were found to be of high quality. All of these high quality studies reported a significant association between depression and knee pain and thus there is strong evidence for this relationship. (level of evidence: strong).

#### Anxiety and knee pain

A nested case control study and longitudinal cohort study, both of high quality, found no association between anxiety and knee pain. Thus we conclude that there is evidence for no association between anxiety and knee pain (level of evidence: limited).

#### Poor mental health and knee pain

While there were four cross-sectional studies that examined the relationship between poor mental health and knee pain, only two were of high quality and both of these found no evidence of a relationship between poor mental health and knee pain. Thus there is evidence for no relationship between poor mental health and knee pain (level of evidence: minimal).

## Discussion

In this systematic review we found strong evidence for a relationship between depression and knee pain, limited evidence that there is no association between anxiety and knee pain and minimal evidence suggesting there is no relationship between poor mental health and knee pain. These results highlight the important role of psychological functioning in knee pain and the need for a biopsychosocial approach to the management of this disabling condition.

We found strong evidence for a positive association between depression and knee pain in adults. This included evidence from 3 RCTs that showed treatment with antidepressant medication was associated with significant pain reduction. The emerging evidence on pathogenesis of depression suggests that it is associated with dysfunction in the inflammatory cytokine production as a response to stressors [45], dysregulation of autonomic nervous system [46,47] and destabilising effect on hypothalamic-pituitary-adrenal axis [48]. Each of these mechanisms also contributes to the provocation of chronic pain syndrome [46,49,50]. In addition, the noradrenaline and serotonin neurotransmitters, which are involved in the pathophysiology of depression [46], have been shown to have significant roles in endogenous pain inhibitory pathways [51,52]. These findings indicate that physiological similarities exist between depression and chronic pain [47]. Another explanation for the association between depression and knee pain may be via reduced physical activity which could be due to either fear of pain [53] or as a consequence of depression [54]. The resulting muscle wasting and reduced joint stability resulting from less activity may have a negative effect on function and disease outcomes of OA [55,56].

Although there was strong evidence for a relationship between depression and knee pain, we found limited evidence for no association between anxiety and knee pain. A major limitation in examining these studies is the lack of longitudinal data, with only one high quality longitudinal study and one nested case-control study examining the relationship between anxiety and knee pain. Further investigation to understand the relationship between anxiety and knee pain is needed as recent work suggests that higher anxiety is related to poorer function in patients with knee OA [53,57] and relationships between anxiety and pain exist in older community-based adults, which are both longitudinal and reciprocal in nature [58].

There was minimal evidence for no relationship between poor mental health and knee pain based on two high quality cross-sectional studies. These findings contrast to those of depression, where there was strong evidence for a relationship between depressive symptoms and knee pain, and may have resulted from the use of generic measures to measure mental health compared to the specific instruments used to assess depression. Our finding is consistent with a previous systematic review which also found minimal evidence that better mental health is protective of knee pain in those with knee OA [6]. Understanding the role of general mental health on knee pain continues to be limited by the absence of cohort studies and RCTs, as well as the paucity of high quality data. Further investigation is needed.

Knee pain results in significant disability and a substantial reduction in quality of life [59,60]. Although knee structural abnormalities are associated with knee pain, it is clear that structure alone does not account for knee pain. It has been suggested that psychosocial factors may play an important role in knee pain. However, previous systematic reviews have only found limited evidence for relationships between both depression and poor mental health and knee symptoms [6,7]. Our systematic review, which is the first to our knowledge to focus on the role of psychosocial factors in knee pain, found that depression has an important role in knee pain. Specifically, the three RCTs of depression found that the treatment with the antidepressant duloxetine resulted in a significant reduction in knee pain [41-43] and is ‘proof of concept’ that depression has an important role in knee pain. While pharmacological interventions, such as antidepressants may be important in the management of knee pain, non-pharmacological strategies, including cognitive behavioural therapy, may also play a significant role. Future research, particularly in the form of RCTs, is needed to examine the effectiveness of non-pharmacological treatment options for reducing depression in the treatment of knee pain.

There are several limitations in undertaking this review. Examining the role of psychosocial factors in knee pain is complex and preliminary searches identified a particularly large number of studies examining a variety of psychosocial factors. We were therefore required to narrow our review to depression, anxiety and general mental health, closely related psychological constructs, which means that there are psychosocial factors that are potentially important in the development of knee pain that we have not investigated. Moreover, while depression, anxiety and general mental health were considered separately and could not be combined due to measurement factors, it is important to note that there is potential overlap between these psychosocial factors.

Moreover, we were not able to perform a meta-analysis to summarize our results due to the heterogeneity of the studies included in this review, and therefore, a best-evidence synthesis was performed. Another limitation was the lack of high quality cohort and RCTs investigating poor mental health and anxiety as risk factors for knee pain. The majority of studies in this review were cross-sectional or case-control studies which limited the quality of the evidence. Another methodological issue identified was the lack of double-blinded assessment of participants which reduced the quality of the data. Furthermore, there was significant heterogeneity in terms of the instruments used to assess the psychological factors.

## Conclusions

This systematic review found that psychological functioning plays an important role in knee pain, with strong evidence for depression being associated with knee pain. We also found limited evidence for anxiety having no relationship with knee pain and minimal evidence for no relationship between poor mental health and knee pain. This review highlights the need for a biopsychosocial approach, in particular addressing psychosocial factors such as depression, in optimising outcomes for knee pain. This is important given the increasing understanding of the complexity of knee pain and potential complications arising from many of the treatments in current use. A holistic approach to managing knee pain has the potential to improve patient outcomes.

## Abbreviations

OA: Osteoarthritis; MRI: Magnetic resonance imaging; RCT: Randomised controlled trial; PRISMA: Preferred Reporting Items for Systematic Reviews and Meta-analysis; WOMAC: Western Ontario and McMaster Universities Arthritis Index; SNRI: Serotonin noradrenalin reuptake inhibitor.

## Competing interests

The authors declare that they have no competing interest.

## Authors’ contributions

PP was involved in data extraction and interpretation and manuscript preparation. JD was involved in acquisition of data, data extraction and manuscript preparation. FC contributed to conception/design, interpretation of data, and manuscript preparation. SF contributed to acquisition of data, data extraction and manuscript preparation. PR was involved in acquisition of data and manuscript preparation. AW and YW contributed to analysis and interpretation of data and manuscript preparation. DU contributed to conception/design, data interpretation and manuscript preparation. All authors read and approved the final manuscript.

## Authors’ information

Pyae Phyomaung and Julia Dubowitz: Joint first authors.

## Pre-publication history

The pre-publication history for this paper can be accessed here:

http://www.biomedcentral.com/1471-2474/15/10/prepub

## Supplementary Material

Additional file 1 Methodological Quality Assessment. Click here for file
